# ZNF281 drives hepatocyte senescence in alcoholic liver disease by reducing HK2‐stabilized PINK1/Parkin‐mediated mitophagy

**DOI:** 10.1111/cpr.13378

**Published:** 2022-12-14

**Authors:** Chunfeng Lu, Ting Ge, Yunyun Shao, Wenqian Cui, Zhe Li, Wenxuan Xu, Xiaofeng Bao

**Affiliations:** ^1^ School of Pharmacy Nantong University Nantong China; ^2^ School of Life Science and Technology China Pharmaceutical University Nanjing China

## Abstract

We investigated the role of zinc‐finger protein 281 (ZNF281), a novel molecule, in ethanol‐induced hepatocyte senescence and uncovered the potential mechanism. Real‐time PCR, Western blot, immunofluorescence staining, and enzyme‐linked immunosorbent assay were performed to explore the role of ZNF281 in hepatocyte senescence. ZNF281 expression was upregulated in both alcohol‐fed mice livers and ethanol‐treated hepatocytes. Silence of ZNF281 in hepatocytes using siRNA mitigated ethanol‐caused decrease in cell viability and increased release of aspartate aminotransferase, alanine transaminase, and lactate dehydrogenase. ZNF281 siRNA reduced senescence‐associated β‐galactosidase‐positive cells under ethanol exposure, abolished cell cycle arrest at G0/G1 phase, and diminished senescence‐associated secretory phenotype and proinflammatory cytokines (IL‐1β and IL‐6) release. At molecular level, ZNF281 deficiency altered the expression profile of senescence‐associated proteins including p53, p21, p16, high mobility group AT‐hook 1, and phospho‐histone H2A.X and telomerase‐associated regulatory factors including telomerase reverse transcriptase, telomeric repeat binding factor 1 (TRF1), and TRF2. ZNF281 knockdown promoted hepatocyte recovery from ethanol‐induced mitochondrial dysfunction and ROS production, which was correlated with rescuing HK2‐PINK1/Parkin signalling‐mediated mitophagy. Mechanistically, ZNF281 directly bound to 5′‐GGCGGCGGGCGG‐3′ motif within HK2 promoter region and transcriptionally repressed HK2 expression. Systematic ZNF281 knockdown by adeno‐associated virus encoding ZNF281 shRNA protected mice from alcohol feeding‐caused hepatocyte injury and senescence. This study provides a novel factor ZNF281 as a driver of hepatocyte senescence during alcoholic liver disease.

## Graphical abstract

Under physiological conditions, damaged mitochondria can be removed and recycled via PINK1/Parkin‐mediated mitophagy, maintaining cellular homeostasis. Under pathological conditions like ethanol exposure, electron transport chain (ETC) breaks are enhanced and mitochondrial membrane potential is declined, inducing oxidative stress and lipid peroxidation. Ethanol also induces ZNF281 expression in hepatocytes, which translocates to nucleus to serve as a transcriptional repressor of HK2. The absence of HK2 decreased PINK1‐Parkin interaction and mitophagy process, causing accumulation of damaged mitochondria. Afterwards, cellular senescence was provoked with the characteristics of cell cycle arrest and senescence‐associated secretory phenotype, promoting the progression of alcoholic liver disease.

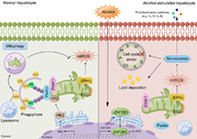

## 1 INTRODUCTION

Alcoholic liver disease is a type of liver disease caused by heavy alcohol consumption, which contains a wide range of hepatic disorders, such as asymptomatic steatosis, alcoholic steatohepatitis, alcoholic liver fibrosis and cirrhosis, and alcohol‐related hepatocellular carcinoma (HCC).^1^ In Western countries, alcoholic liver disease is identified as a primary pathogenic factor for liver cirrhosis.^2^ In China, the incidence of alcoholic liver disease increases by years due to more beverage consumption under improved living standards.^3^ Alcoholic liver disease has been become a universal public health issue and imposed a heavy burden to national economy. However, the molecular pathology underlying the occurrence and development of alcoholic liver disease has not been fully revealed. Thus, it is of great urgence and importance to lucubrate the possible pathological mechanisms, which may provide a statistical basis for the development of potential therapeutic strategy.

Cellular senescence is commonly existed in multiple physiological and pathological events. Physiologically, cell senescence and regeneration contribute to the renewal of body tissues and organs and the maintenance of life activities.^4^ Morphologically, cells undergoing senescence are enlarged and flattened with swollen nuclei. Senescent cells are also featured by low proliferation capacity, senescence‐associated β‐galactosidase (SA‐β‐gal) positivity, cell cycle arrest, and telomere shortening.^5^ Most senescent cells may exhibit a senescence‐associated secretory phenotype (SASP) and overproduce massive proinflammatory cytokines, aggravating inflammation and related pathology.^6^ Cellular senescence could be driven by various factors, including genotoxic stress, oxidative stress, inflammatory cytokines, etc.^7^ Previous studies have revealed that alcohol as well as its endogenous metabolite acetaldehyde induced cellular oxidative stress and DNA double strand breaks.^8^ Notably, our studies further specified that hepatocytes under chronic alcohol exposure were manifested with senescence‐like cell appearance and behaviour, which accelerates the progression of alcoholic liver disease.^5^, ^6^, ^9^ However, according to currently available studies, the node factors that comprehensively regulate the process of senescence are far from clear.

Zinc‐finger protein 281 (ZNF281), also known as ZFP281/ZBP‐99, is a 99 kDa zinc‐finger transcriptional regulator that binds to GC‐rich regions located in the promoters of a variety of genes.^10^ ZNF281 has been widely reported to be associated with the progression of many cancers.^11^, ^12^ In HCC, ZNF281 was found be to aberrantly upregulated and promoted HCC invasion and migration,^13^ which was a preliminary reflection of the potential correlation between ZNF281 and liver disease. Intriguingly, ZNF281 expression was increased in human osteosarcoma cells under genotoxic stress caused by etoposide, a DNA‐damaging as well as senescence‐induced agent; Mechanistically, ZNF281 was recruited to the sites of DNA damage and contributed to DNA damage response by transcriptionally regulating several regulatory factors.^14^ These findings suggested that ZNF281 could be associated with cellular senescence, but whether and how ZNF281 could be involved in cellular senescence, hepatocytes to be specific, need to be clarified. Dysfunctional mitochondria, which can be removed by macroautophagy/mitophagy, were found to be accumulative in hepatocytes that develop a senescence‐like phenotype in response to persistent DNA damage.^15^ ZNF281 was reported to be involved in the regulation of autophagy process in UCA1‐mediated 5‐fluorouracil resistance of colorectal cancer cells.^16^ These findings suggested that ZNF281 may participate in the modulation of cellular senescence and this could be associated with autophagy regulation.

In this work, we used both in vivo and in vitro models to mimic clinical alcoholic liver disease to study the pathogenic role of ZNF281 and potential mechanisms. According to our results, we identified that ZNF281 expression was abnormally elevated in alcohol‐exposed murine livers and hepatocytes under ethanol exposure. Silence of ZNF281 expression in hepatocytes rescued ethanol‐caused hepatocyte injury and senescence‐like phenotype. Mechanistically, ZNF281 could bind to the 5′ promoter region of hexokinase II (HK2) gene and transcriptionally repress HK2 expression, which impedes HK2‐mediated PINK1/Parkin signalling initiation and retarded mitophagy. In vivo systematic knockdown of ZNF281 relieved alcohol‐caused liver damage in mice. In summary, our study for the first time reveals the pathogenic role of ZNF281 in alcoholic liver disease and elucidates a potential molecular mechanism, which may provide a novel insight into the pathophysiological researches of alcoholic liver disease.

## 2 MATERIALS AND METHODS

### 2.1 Antibodies and reagents

Primary antibodies against ZNF281, p53, p21, p16, telomerase reverse transcriptase (TERT), telomeric repeat binding factor 2 (TRF2), hexokinase II (HK2), and β‐actin were purchased from Santa Cruz Biotechnology (Santa Cruz, CA, USA). Primary antibody against high mobility group AT‐hook 1 (HMGA1) was purchased from Biorbyt (Cambridge, UK). Primary antibodies against phospho‐histone H2A.X (γH2A.X) and TRF1 were purchased from Beijing Bioss Biotechnology Co., Ltd. (Beijing, China). Primary antibodies against light chain 3 (LC3), p62, PTEN‐induced putative kinase 1 (PINK1), Parkin, and translocase of outer mitochondrial membrane 20 (TOM20) were purchased from Proteintech Group, Inc. (Rosemont, IL, USA). Horseradish peroxidase‐conjugated and fluorescein‐conjugated secondary antibodies were purchased from Cell Signaling Technology (Danvers, MA, USA). Mito‐Tracker Red CMXRos, Lyso‐Tracker Green, and SA‐β‐gal staining kit were purchased from Beyotime Biotechnology (Shanghai, China). MitoSOX Red mitochondrial superoxide indicator, Nile Red, and DAPI were purchased from Yeasen Biotechnology Co., Ltd. (Shanghai, China). Cell counting kit‐8 (CCK‐8) and mitochondria isolation kit were purchased from Beyotime Biotechnology. Aspartate aminotransferase (AST), alanine transaminase (ALT), lactate dehydrogenase (LDH), triglyceride (TG), and total cholesterol (TC) assay kits were purchased from Nanjing Jiancheng Bioengineering Institute (Nanjing, Jiangsu, China). IL‐1β, IL‐6, interferon gamma (IFNγ), and tumour necrosis factor alpha (TNFα) enzyme‐linked immunosorbent assay (ELISA) kits were purchased from Nanjing SenBeiJia Biological Technology Co., Ltd. (Nanjing, Jiangsu, China). Cell cycle detection kit was purchased from KeyGEN BioTECH (Nanjing, Jiangsu, China). Dual‐luciferase reporter system was purchased from Promega (Madison, WI, USA). Recombinant ZNF281 plasmid (pcDNA3.1(+)‐ZNF281) and HK2 promoter luciferase reporter plasmid (pGL3‐HK2‐wt) were constructed using molecular cloning technology. ZNF281 shRNA (shZNF281) and negative control shRNA (shNC) were purchased from Santa Cruz Biotechnology and encoded using adeno‐associated virus vectors by Nanjing Dirui Biological Technology Co., Ltd. (Nanjing, Jiangsu, China). ZNF281 siRNA (siZNF281), HK2 siRNA (siHK2), and negative control siRNA (siNC) were purchased from Santa Cruz Biotechnology. Lipofectamine 2000 reagent was purchased from Sigma‐Aldrich (St. Louis, MO, USA). Primers designed for quantitative real‐time PCR (qRT‐PCR), chromatin immunoprecipitation (ChIP)‐qRT‐PCR, and site‐directed mutagenesis were synthesized by Hongxun Biotechnology (Suzhou, Jiangsu, China) and listed in Tables [Link], [Link]. Site‐directed mutagenesis kit was purchased from Sangon Biotech (Shanghai, China). ChIP assay kit was purchased from Beyotime Biotechnology.

### 2.2 Animal experiments

#### 2.2.1 Animals

Seventy‐two male ICR mice, weighed 20–25 g, were purchased from Nantong University (Nantong, Jiangsu, China). Mice were raised in cages and housed in a specific pathogen‐free room with a constant temperature interval during 21–25°C and a 12‐h light/12‐h dark cycle. All mice were fed on a standard chow diet and received human care according to the National Institutes of Health Guidelines of People's Republic of China. The animal experiments were approved by the Institutional and Local Committee on the Care and Use of Animals of Nantong University.

#### 2.2.2 Study I

Thirty‐six mice were randomly assigned into three groups (12 mice per group). Mice in alcohol‐treated groups were intragastrically administrated with alcohol (56% v/v, 10 ml/kg) once daily for consecutive 2 or 4 weeks. Mice in vehicle group were intragastrically administrated with normal saline once daily.

#### 2.2.3 Study II

Thirty‐six mice were randomly divided into three groups (12 mice per group). Mice were infected with adenovirus‐associated virus vectors encoding shNC or shZNF281 (1 × 10^10^ genomic copies per mice) by caudal vein injection on the first day. Then, mice were intragastrically administrated with alcohol (56% v/v, 10 ml/kg) or normal saline once daily for consecutive 4 weeks.

At 24 h after the last administration, blood was collected by orbital vein. Murine liver tissues were harvested and stored in liquid nitrogen for further analysis.

### 2.3 Cell culture and treatment

Human normal hepatocytes HL‐7702 cells were purchased from Cell Bank of Chinese Academy of Sciences (Shanghai, China). Cells were cultured in Dulbecco's modified eagle medium (DMEM; Invitrogen, Grand Island, NY, USA) supplemented with 10% fetal bovine serum (FBS; Gibco, Invitrogen), 100 U/ml of penicillin, and 100 μg/ml of streptomycin (Beyotime Biotechnology). Cells were incubated under a constant temperature of 37°C and a humidified atmosphere of 95% air and 5% CO_2_. Cells were administrated with different reagents of different concentrations for indicated time before experimental processing.

### 2.4 Transient transfection

Recombinant plasmid or siRNA was mixed with 150‐μl serum‐free and antibiotics‐free culture medium. Seven microlitres of lipofectamine 2000 reagent was mixed with additional 150‐μl culture medium. The two mixtures were dividually incubated at room temperature for 5 min, then gently blended, and incubated at room temperature for 10 min. Seven hundred microlitres of culture medium was added into the system and 1‐ml transfection mixture was obtained. For transfection, cells in each well of six‐well plates were incubated with 1‐ml transfection mixture for 24 or 48 h.

### 2.5 Immunoblot assay

Murine liver tissues and human hepatocytes were lysed using radioimmunoprecipitation assay buffer, phenylmethylsufonyl fluoride (PMSF), and phosphatase inhibitor for extracting total proteins. Protein concentrations were detected using a BCA assay kit (Beyotime Biotechnology). Proteins were separated by electrophoresis using sodium dodecyl sulphate‐polyacrylamide gels (SDS‐PAGE) and then transferred to polyvinylidene fluoride membranes (Millipore, Burlington, MA, USA). The membranes were incubated with tris‐buffered saline containing 0.1% Tween 20 and 5% skim milk at room temperature for 1 h for blocking non‐specific binding. The membranes were incubated with specific primary antibodies overnight at 4°C and then secondary antibodies at room temperature for 1 h. Protein bands were visualized using chemiluminescence reagents (Millipore) and densitometrically detected using ImageQuant LAS 4000 equipped with ImageQuant TL 7.0 Software (GE Healthcare, New Orleans, LA, USA).

### 2.6 RNA extraction and qRT‐PCR assay

Total RNA in murine liver tissues and HL‐7702 hepatocytes was extracted using Trizol reagent according to the protocol from manufacturer (Sigma‐Aldrich). qRT‐PCR analysis was performed according to our previous description with modification.^17^ Glyceraldehyde phosphate dehydrogenase (GAPDH) was used as an invariant control, and mRNA levels were expressed as fold changes after normalization to GAPDH.

### 2.7 Fluorescence staining

Immunofluorescence staining for visualizing interested proteins were performed according to our previous description with modification.^18^ Mitochondria and lysosomes were dividually labelled using Mito‐Tracker Red CMXRos and Lyso‐Tracker Green probes according to the protocols provided by manufacturer. Lipid droplets were stained by Nile Red dye according to the instruction from manufacturer. Mitochondrial ROS production was measured using mitochondrion‐localized probe MitoSOX Red. Fluorescent images were taken at 63× magnification with oil‐immersion using a Leica laser scanning confocal microscope (Leica, Wetzlar, Germany).

### 2.8 CCK‐8 assay

Cell viability was detected by CCK‐8 assay according to the protocol provided by manufacturer. The absorbance values were recorded using a SPECTRAmax™ microplate spectrophotometer (Molecular Devices, Sunnyvale, CA, USA).

### 2.9 Biochemical analysis

The activities of AST, ALT, and LDH in cell culture supernatant or serum and the levels of intracellular TG and TC were measured using commercial assay kits. The levels of IL‐1β, IL‐6, IFNγ, and TNFα in cell culture supernatant or serum were determined using ELISA kits according to the protocols from manufacturer. The absorbance values were determined using a SPECTRAmax™ microplate spectrophotometer.

### 2.10 SA‐β‐gal staining

Cells were fixed with 4% paraformaldehyde for 15 min, washed with phosphate buffer saline (PBS) containing 1 mM MgCl_2_, and stained overnight with PBS containing 1 mM MgCl_2_, 1 mg/ml X‐gal, and 5 mM potassium ferricyanide (pH = 6.0). Images were taken using a light microscope.

### 2.11 Isolation of mitochondria

Mitochondria were isolated from hepatocytes using a mitochondrion isolation kit according to the manufacturer's instruction. Briefly, cells were homogenized in ice‐cold mitochondria isolation buffer with 1‐mM PMSF and centrifuged at 1000*g* for 10 min at 4°C. The supernatants were collected in a new centrifuge tube and centrifuged at 3500*g* for 10 min at 4°C. The sediment was collected as mitochondria. The supernatant was centrifuged at 12000*g* for 10 min at 4°C for collecting cytoplasmic fraction.

### 2.12 Co‐immunoprecipitation (Co‐IP) assay

Cells were lysed by 50‐mM Tris–HCl buffer (pH 7.4) containing 150‐mM NaCl, 1‐mM EDTA, 0.5% nonidet P‐40, and protease inhibitors. Cell lysates with a protein concentration of 1 μg/μl were incubated by primary antibody against PINK1 or Parkin. After an overnight gentle rocking at 4°C, Dynabeads® protein G (Invitrogen) and the lysate/antibody mixture were added into one tube and co‐incubated with vortex at 4°C for 4 h. The tube containing immunoprecipitants was placed on a magnet. Then, the supernatant was discarded. The immunoprecipitants were washed for three times and heated at 70°C for 10 min in the presence of elution buffer, premixed NuPAGE® LDS sample buffer, and NuPAGE sample reducing agent. Precipitated proteins were separated by SDS‐PAGE and subjected to immunoblot analysis.

### 2.13 Dual‐luciferase reporter assay

Treated cells were co‐transfected with recombinant promoter luciferase reporter plasmid and internal reference plasmid pRL‐TK for 48 h. Luciferase activities were measured using a dual‐luciferase reporter assay system (Promega) according to the protocol from manufacturer and presented as folds of control after normalization to *Renilla* luciferase activities.

### 2.14 Bioinformatics analysis and site‐directed mutagenesis

The binding sites of ZNF281 to human *HK2* promoter region were predicted using JASPAR database. In short, 2000 bp of *hHK2* gene regulatory region (−2000 to 0 bp, transcription start site [TSS] = +1) was extracted from Esembl and used to search ZNF281 sites with a matrix (MA1630.2). A relative score cutoff of 0.8 was used to identify putative sites. Transcript used for this analysis was *hHK2* ENSG00000159399. The putative binding sites were, respectively, mutated using a site‐directed mutagenesis kit. Mutated PCR products were inserted between XhoI and Hind sites of pGL3‐basic luciferase reporter plasmid vector.

### 2.15 ChIP‐qRT‐PCR assay

ChIP assay was performed using a ChIP assay kit according to the manufacturer's instruction. Briefly, cells were cross‐linked with 1% formaldehyde. A total of 200–1000 bp DNA fragments were obtained by sonication. ChIP‐grade ZNF281 antibody and non‐specific IgG mouse antibody was precipitated with chromatin overnight at 4°C. Then DNA was extracted and purified from the chromatin complexes and HK2 abundance were analysed by qRT‐PCR.

### 2.16 Statistical analysis

Data were analysed using Graphpad Prism Software Version 5.0 (Graphpad Software, La Jolla, CA, USA) and presented as mean ± SD. The significance of difference was determined by Student's *t*‐test (comparison between two groups) and one‐way analysis of variance (ANOVA) with *post*‐*hoc* Tukey test (comparison between three or more groups). The values of *p* < 0.05 were considered statistically significant.

## 3 RESULTS

### 3.1 ZNF281 expression is upregulated in both in vivo and in vitro alcoholic liver disease models

**FIGURE 1:**
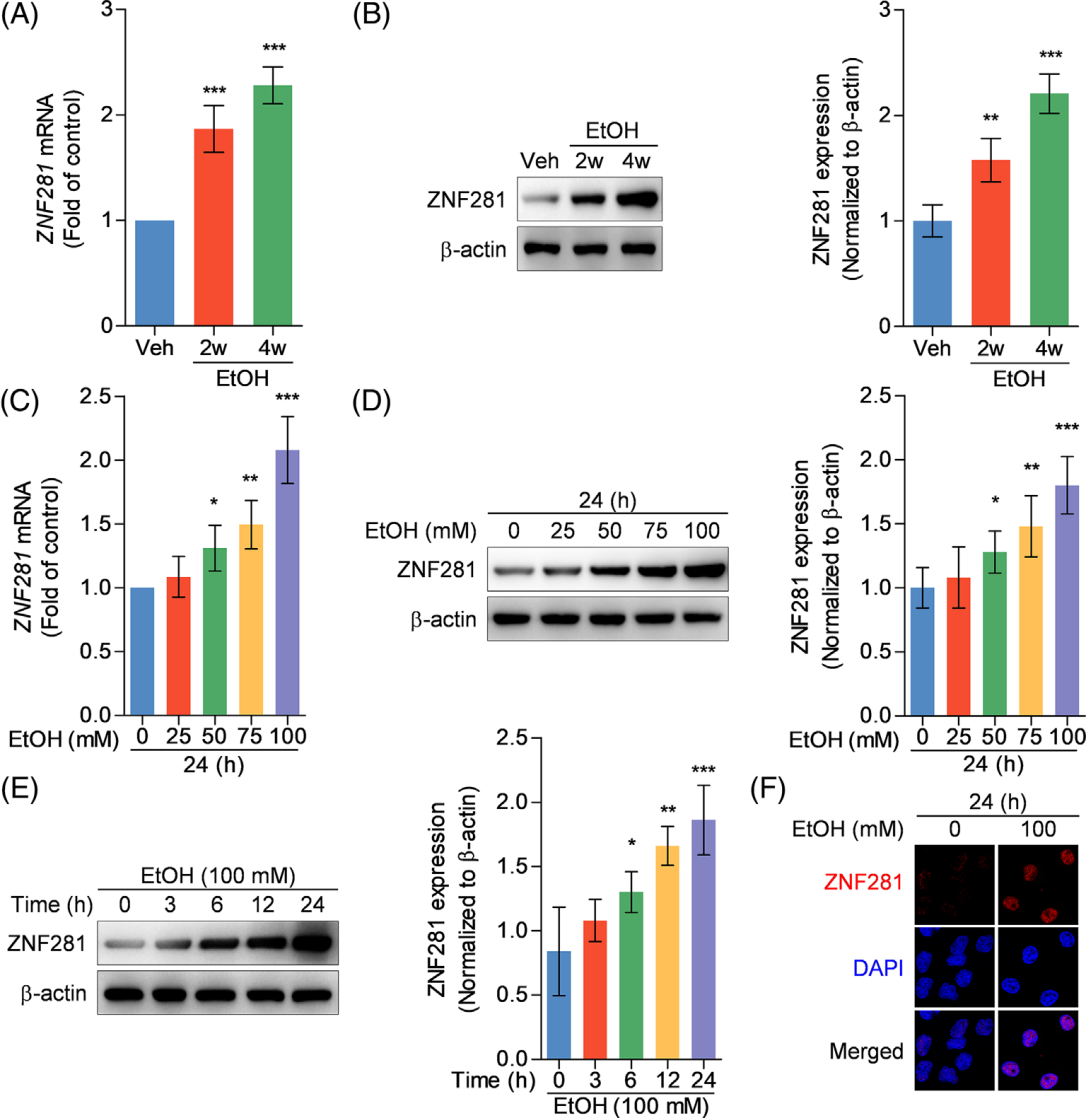
ZNF281 expression is upregulated in both in vivo and in vitro alcoholic liver disease models. (A, B) Mice were intragastrically administrated with vehicle or ethanol for consecutive 2 or 4 weeks. Hepatic ZNF281 mRNA and protein abundance measured by qRT‐PCR (A) and immunoblots (B). **p < 0.01, ***p < 0.001 versus vehicle. (C, D) HL‐7702 cells were treated with ethanol with concentrations ranging from 0 to 100 mM for 24 h. Intracellular ZNF281 mRNA and protein abundance measured by qRT‐PCR (C) and immunoblots (D). *p < 0.05, **p < 0.01, ***p < 0.001 versus 0‐mM ethanol for 24 h. (E) HL‐7702 cells were treated with 100‐mM ethanol for time periods ranging from 0 to 24 h. Intracellular ZNF281 protein abundance measured by immunoblots. *p < 0.05, **p < 0.01, ***p < 0.001 versus 100‐mM ethanol for 0 h. (F) HL‐7702 cells were treated with vehicle or 100‐mM ethanol for 24 h. Intracellular ZNF281 protein visualized by immunofluorescence staining

Our previous studies have repeatedly confirmed the rationality and stability of in vivo and in vitro alcoholic liver disease models that we established.^5^, ^6^, ^19^, ^20^ Based on the model, this study showed that both mRNA and protein abundance of ZNF281 in murine liver tissues were progressively increased during alcoholic liver disease (Figure 1A,B). Hepatocytes are the dominant type of liver cells as well as the place for alcohol metabolism wherein metabolic byproducts usually cause hepatocellular damage. To uncover the alternation of ZNF281 in hepatocytes under ethanol stimulation, human hepatocytes HL‐7702 were incubated with ethanol to mimic alcohol‐induced hepatocyte injury. Immunoblots showed that ZNF281 expression was enhanced in a concentration‐dependent manner at both transcriptional and translational levels (Figure 1C,D). Furthermore, 100‐mM ethanol administration stimulated ZNF281 protein expression in a time‐dependent manner (Figure 1E). Immunofluorescence staining showed increased ZNF281 protein in hepatocytes after ethanol exposure, which was located in the nuclei region (Figure 1F). Collectively, these data indicated that ZNF281 expression positively correlates with the progress of alcoholic liver disease.

### 3.2 ZNF281 deficiency alleviates ethanol‐caused hepatotoxicity

**FIGURE 2:**
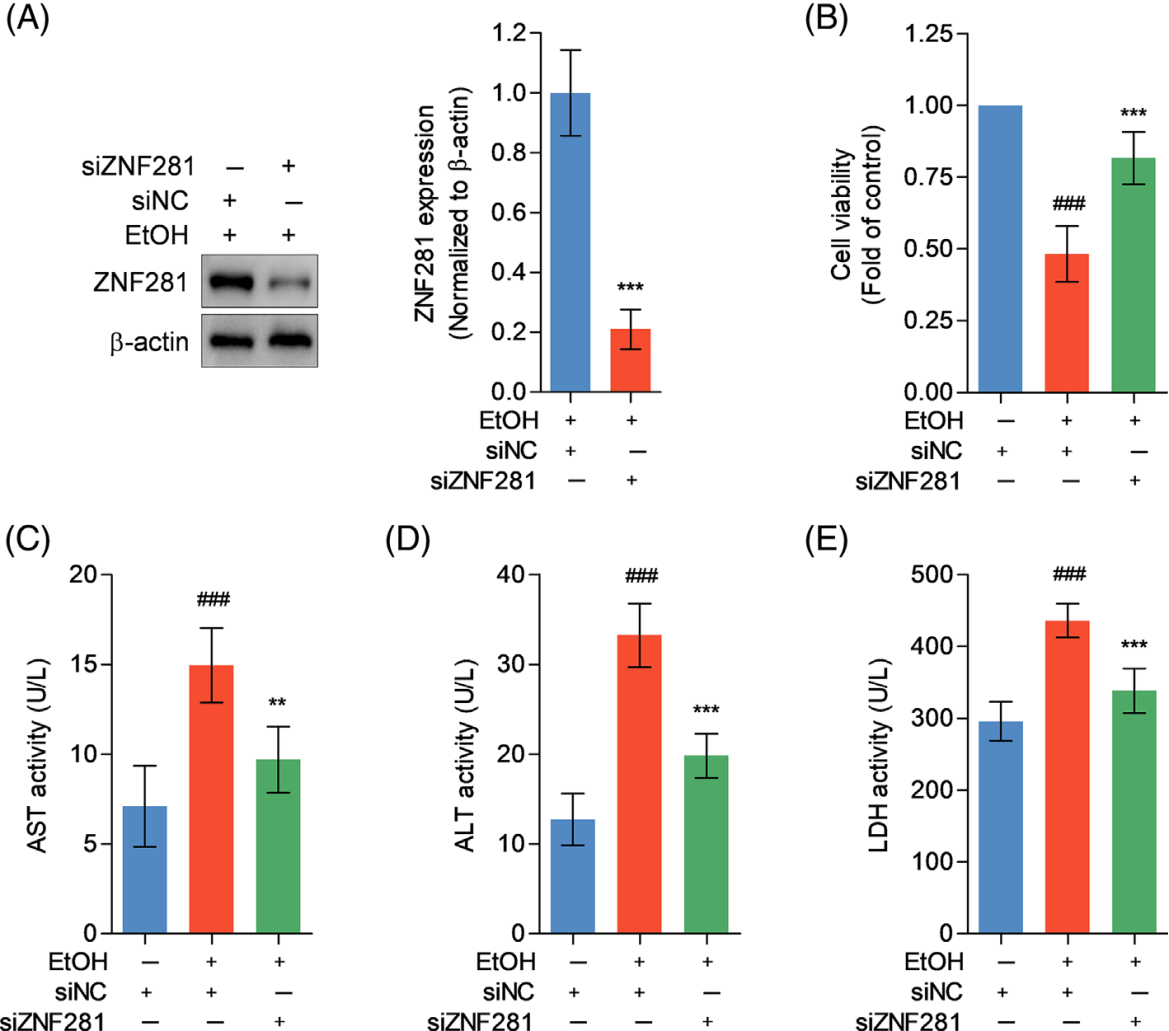
ZNF281 deficiency alleviates ethanol‐caused hepatotoxicity. (A) HL‐7702 cells were transfected with siNC or siZNF281 for 24 h and treated with 100‐mM ethanol for 24 h. Intracellular ZNF281 protein abundance measured by immunoblots. ***p < 0.001 versus siNC+100‐mM ethanol. (B–E) HL‐7702 cells were transfected with siNC or siZNF281 for 24 h and treated with vehicle or 100‐mM ethanol for 24 h. (B) Cell viability detected by CCK‐8. (C–E) AST, ALT, and LDH levels in cell culture supernatant. ^###^ *p* < 0.001 versus siNC+vehicle. **p < 0.01, ***p < 0.001 versus siNC+100‐mM ethanol

To investigate whether increased ZNF281 protein mediated hepatocyte injury under ethanol exposure, siRNA was applied to silence ZNF281 expression. Immunoblots verified the efficacy of ZNF281 siRNA in diminishing ethanol induction on ZNF281 expression in hepatocytes (Figure 2A). CCK‐8 assay showed that 100‐mM ethanol administration for 24 h significantly damaged cell viability, which was rescued by ZNF281 knockdown (Figure 2B). Ethanol stimulation caused cellular injury evidenced by increased enzyme activities of AST, ALT, and LDH in culture supernatant of control hepatocytes but not ZNF281‐knockdown hepatocytes (Figure 2C–E). Taken together, these results indicated that ZNF281 knockdown alleviates ethanol‐induced hepatotoxicity.

### 3.3 ZNF281 deficiency reduces hepatocyte senescence under ethanol exposure

**FIGURE 3:**
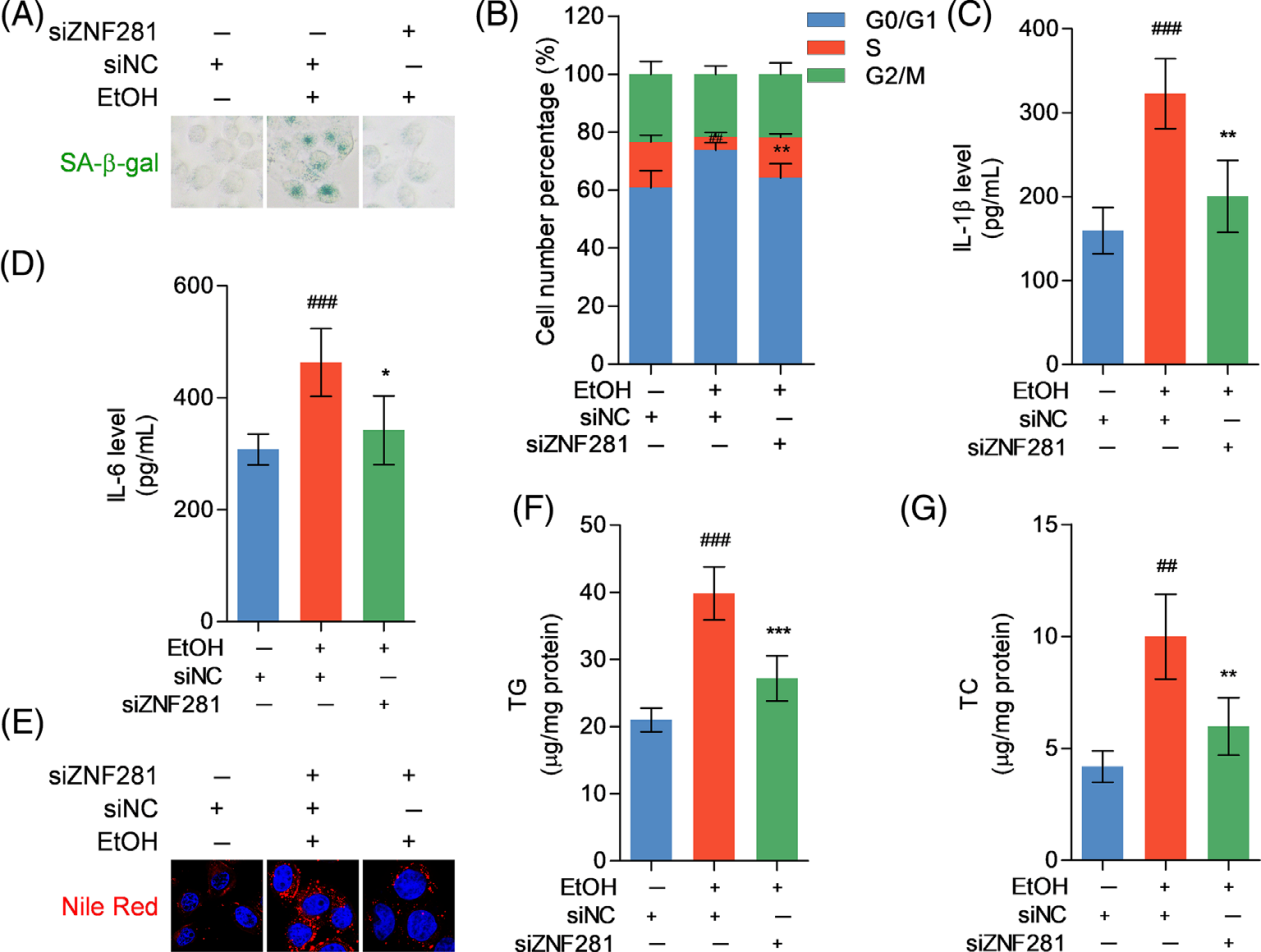
ZNF281 deficiency reduces hepatocyte senescence under ethanol exposure. HL‐7702 cells were transfected with siNC or siZNF281 for 24 h and treated with vehicle or 100‐mM ethanol for 24 h. (A) Senescent cells labelled by SA‐β‐gal staining. (B) The number of cells in different cell cycles detected by flow cytometry. (C, D) IL‐1β and IL‐6 levels in cell culture supernatant determined by ELISA. (E) Intracellular lipid droplets stained by Nile Red dye. (F, G) Intracellular TG and TC levels. ^##^ *p* < 0.01, ^###^ *p* < 0.001 versus siNC+vehicle. *p < 0.05, **p < 0.01, ***p < 0.001 versus siNC+100‐mM ethanol

SA‐β‐gal staining showed increased SA‐β‐gal‐positive puncta in ethanol‐exposed hepatocytes, which was not observed when ZNF281 knockdown (Figure 3A). Flow cytometry showed that control hepatocytes were arrested at G0/G1 phase by ethanol incubation, while ZNF281‐knockdown hepatocytes were resistant to ethanol insult, released from G0/G1 phase, and entered S phase (Figure 3B). SASP is a critical marker for cellular senescence, which promotes the production of proinflammatory cytokines.^21^ Proinflammatory cytokines IL‐1β and IL‐6 were significantly increased in culture supernatant of ethanol‐treated hepatocytes, while ZNF281 silence reduced the release of IL‐1β and IL‐6 under ethanol stimulation (Figure 3C,D). Senescent hepatocytes are also featured by lipometabolic disorder.^22^ Intracellular deposition of lipid droplets was more evident in control hepatocytes under ethanol exposure when compared with ZNF281‐silent hepatocytes (Figure 3E). TG and TC levels in hepatocytes were elevated when administrated with ethanol but further decreased when co‐administrated with ZNF281 siRNA (Figure 3F,G). Altogether, these findings suggested that ZNF281 deficiency mitigates the induction of ethanol on hepatocyte senescence.

### 3.4 ZNF281 knockdown rescues senescence‐associated genes expression in ethanol‐exposed hepatocytes

**FIGURE 4:**
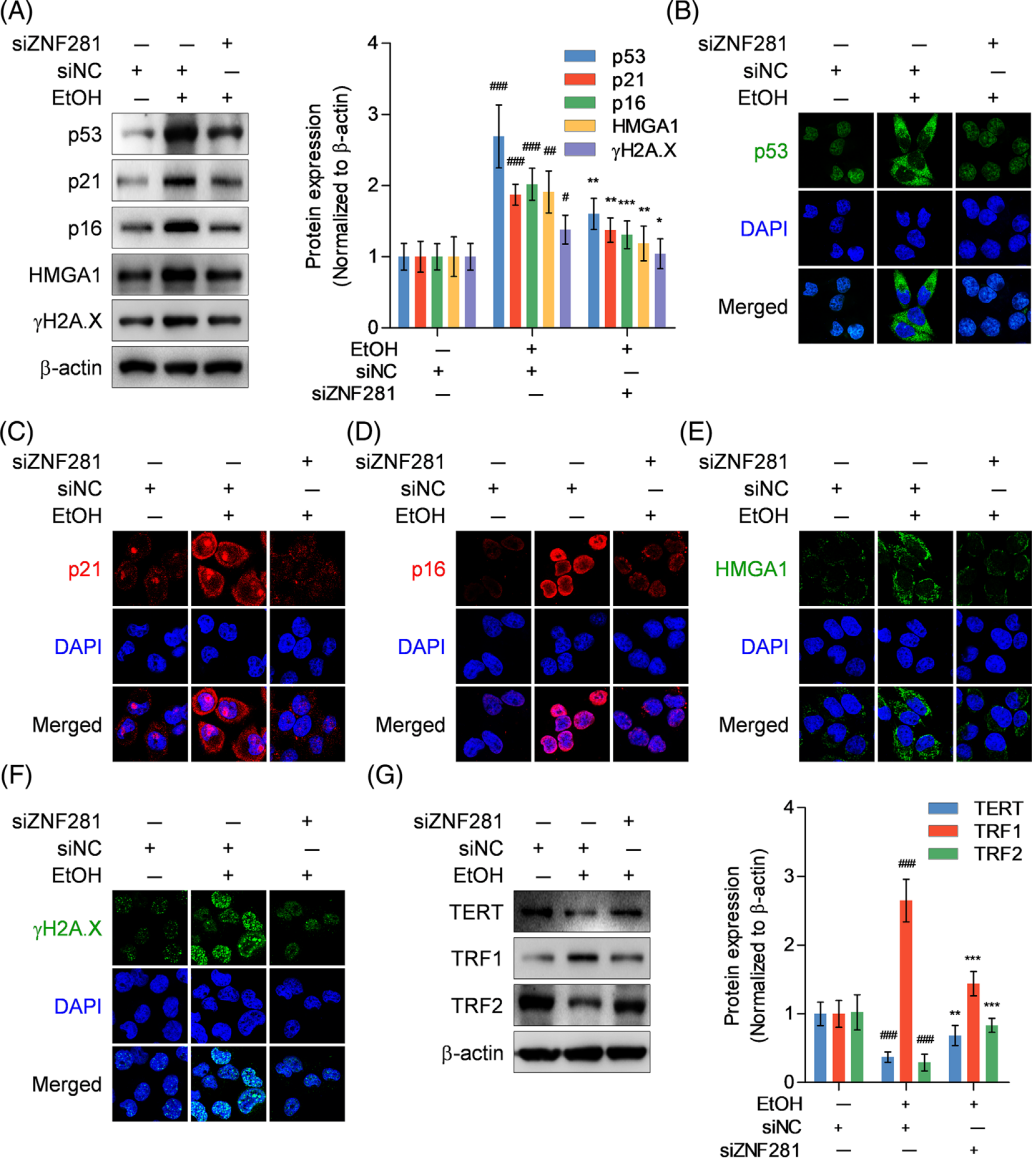
ZNF281 knockdown rescues senescence‐associated genes expression in ethanol‐exposed hepatocytes. HL‐7702 cells were transfected with siNC or siZNF281 for 24 h and treated with vehicle or 100‐mM ethanol for 24 h. (A–F) Intracellular p53, p21, p16, HMGA1, and γH2A.X protein abundance measured by immunoblots and immunofluorescence staining. (G) Intracellular TERT, TRF1, and TRF2 protein abundance measured by immunoblots. ^#^ *p* < 0.05, ^##^ *p* < 0.01, ^###^ *p* < 0.001 versus siNC+vehicle. *p < 0.05, **p < 0.01, ***p < 0.001 versus siNC+100‐mM ethanol

Next, the effect of ZNF281 on ethanol‐caused cellular senescence was explored at molecular level. ZNF281 knockdown negatively regulated the protein expression of p53, p21, and p16 (cell cycle regulators), HMGA1 (senescent cell marker), and γH2A.X (DNA damage marker) in ethanol‐exposed hepatocytes (Figure 4A). Immunofluorescence staining visualized the alternations of protein abundance and subcellular locations. Of note was that p53 was mainly located in cytoplasm of ethanol‐exposed control hepatocytes, differing from normal control hepatocytes, and ZNF281‐knockdown hepatocytes wherein p53 was observed mainly in nuclei (Figure 4B–F). TERT expression is positively correlated with the activity of telomerase.^23^ TRF1 and TRF2 are telomere‐associated proteins that may exert distinguishable impacts on the activity of telomerase.^24^ Immunoblots showed that the expression of TERT and TRF2 was downregulated but TRF1 was upregulated in ethanol‐incubated hepatocytes, which, however, were reversed when ZNF281 knockdown (Figure 4G). In brief, these data demonstrated that ZNF281 facilitates senescent phenotype by regulating the expression pattern of senescent‐associated genes in ethanol‐exposed hepatocytes, partially at least.

### 3.5 ZNF281 knockdown recovers mitophagy in ethanol‐exposed hepatocytes

**FIGURE 5:**
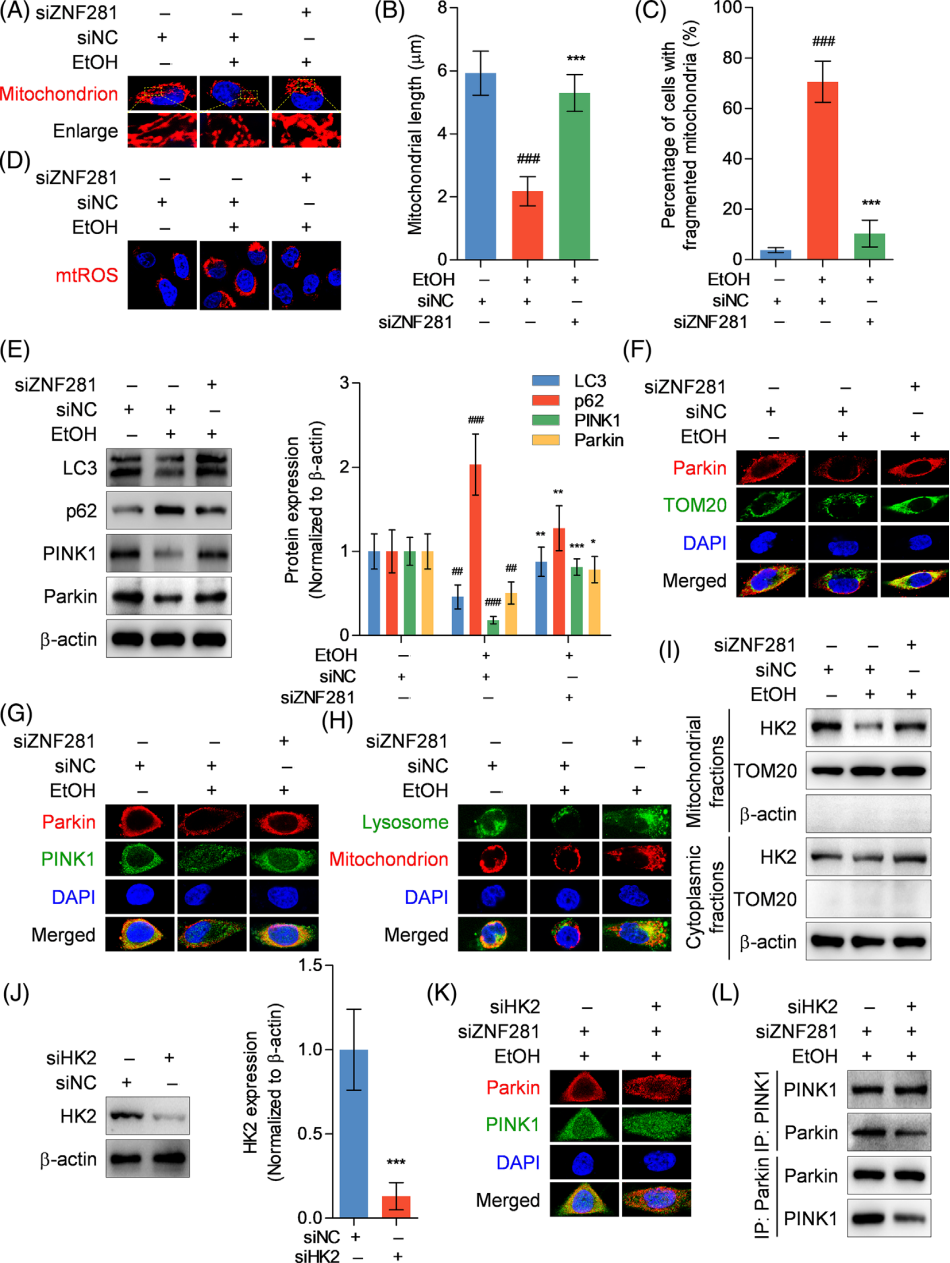
ZNF281 knockdown recovers mitophagy in ethanol‐exposed hepatocytes. (A–I) HL‐7702 cells were transfected with siNC or siZNF281 for 24 h and treated with vehicle or 100‐mM ethanol for 24 h. (A) Mitochondria visualization by Mito‐Tracker probe. (B) Mitochondrial length. (C) Percentage of cells with fragmented mitochondria. (D) Mitochondrial ROS detected by mitoSOX staining. (E) Intracellular LC3, p62, PINK1, and Parkin protein abundance measured by immunoblots. ^##^ *p* < 0.01, ^###^ *p* < 0.001 versus siNC+vehicle. *p < 0.05, **p < 0.01, ***p < 0.001 versus siNC+100‐mM ethanol. (F, G) Colocalization between Parkin and TOM20 or PINK1 visualized by immunofluorescence staining. (H) Mitochondria and lysosomes labelled using Mito‐tracker and Lyso‐Tracker probes. (I) HK2 protein abundance and subcellular location determined by immunoblots. (J) HL‐7702 cells were transfected with siNC or siHK2 for 24 h. Intracellular HK2 protein abundance detected by immunoblots. ***p < 0.001 versus siNC. (K, L) HL‐7702 cells were transfected with siZNF281 or additionally co‐transfected with siHK2 for 24 h and treated with 100‐mM ethanol for 24 h. (K) Colocalization between Parkin and PINK1 visualized by immunofluorescence staining. (L) Interaction between PINK1 and Parkin in hepatocytes detected by Co‐IP

Mitochondrial dysfunction is a pivotal characteristic of hepatocyte injury, which may contribute to cellular senescence. The microstructure and function of mitochondria were analysed between treatments. Mito‐Tracker probe staining showed that ethanol exposure caused mitochondrial fragmentation characterized by organelle cleavage into small spheres or short rods. ZNF281‐knockdown hepatocytes exhibited more normal morphology under ethanol exposure when compared with control hepatocytes (Figure 5A–C). MitoSOX ROS staining for visualizing mitochondrial ROS exhibited that ZNF281 knockdown abolished the inductive effect of ethanol on mitochondrial ROS production (Figure 5D). Mitophagy is a highly‐conserved intracellular metabolic mechanism beneficial to cellular homeostasis via scavenging damaged or aged mitochondria.^15^ Here we aimed to illustrate whether mitophagy pathway mediated ZNF281 modulation of mitochondrial function. Immunoblots indicated that ZNF281 knockdown caused increased LC3 protein abundance and decreased p62 protein abundance in ethanol‐exposed hepatocytes, suggesting maintained autophagic flux in ethanol‐treated ZNF281‐knockdown hepatocytes. Notably, ZNF281 knockdown rescued the protein abundance of PINK1 and Parkin, a dominant cascade initiating mitophagy, in ethanol‐exposed hepatocytes (Figure 5E). Immunofluorescence double staining showed that decreased colocalization between Parkin and TOM20 or PINK1 puncta in ethanol‐incubated hepatocytes, which was reversed when further supplemented with ZNF281 siRNA, suggesting that ZNF281 probably maintained or enhanced recruitment of Parkin to mitochondria or Parkin interaction with PINK1, more exactly (Figure 5F,G). We also observed limited colocalization between lysosomes and mitochondria in ethanol‐incubated hepatocytes, which was rescued by additional ZNF281 knockdown (Figure 5H). HK2 localizes to the outer mitochondrial membrane and serves as an upstream regulator of mitophagy via positively regulating PINK1/Parkin aggregation.^25^ Immunoblots indicated that HK2 protein abundance was decreased in mitochondrial fractions of hepatocytes exposed to ethanol (Figure 5I). Thus, HK2 expression was silenced for exploration on the role of HK2 in ZNF281‐mediated mitophagy inhibition (Figure 5J). Immunofluorescence double staining showed that HK2 deficiency did not alter the abundance of intracellular Parkin and PINK1 but abrogated the effect of ZNF281 siRNA on promoting Parkin‐PINK1 colocalization, suggesting that HK2 mediated PINK1/Parkin pathway activation possibly by affecting molecular interaction and HK2 could be a molecule downstream of ZNF281 regulation (Figure 5K). Co‐IP analysis revealed specific interaction between PINK1 and Parkin in ZNF281‐knockdown hepatocytes with ethanol exposure, which was decreased when co‐transfected with HK2 siRNA (Figure 5L). Therefore, these data supported that ZNF281/HK2 axis is involved in dampening PINK1/Parkin‐mediated mitophagy in hepatocytes under ethanol exposure.

### 3.6 ZNF281 directly binds to HK2 promoter and initiates its transrepression

**FIGURE 6:**
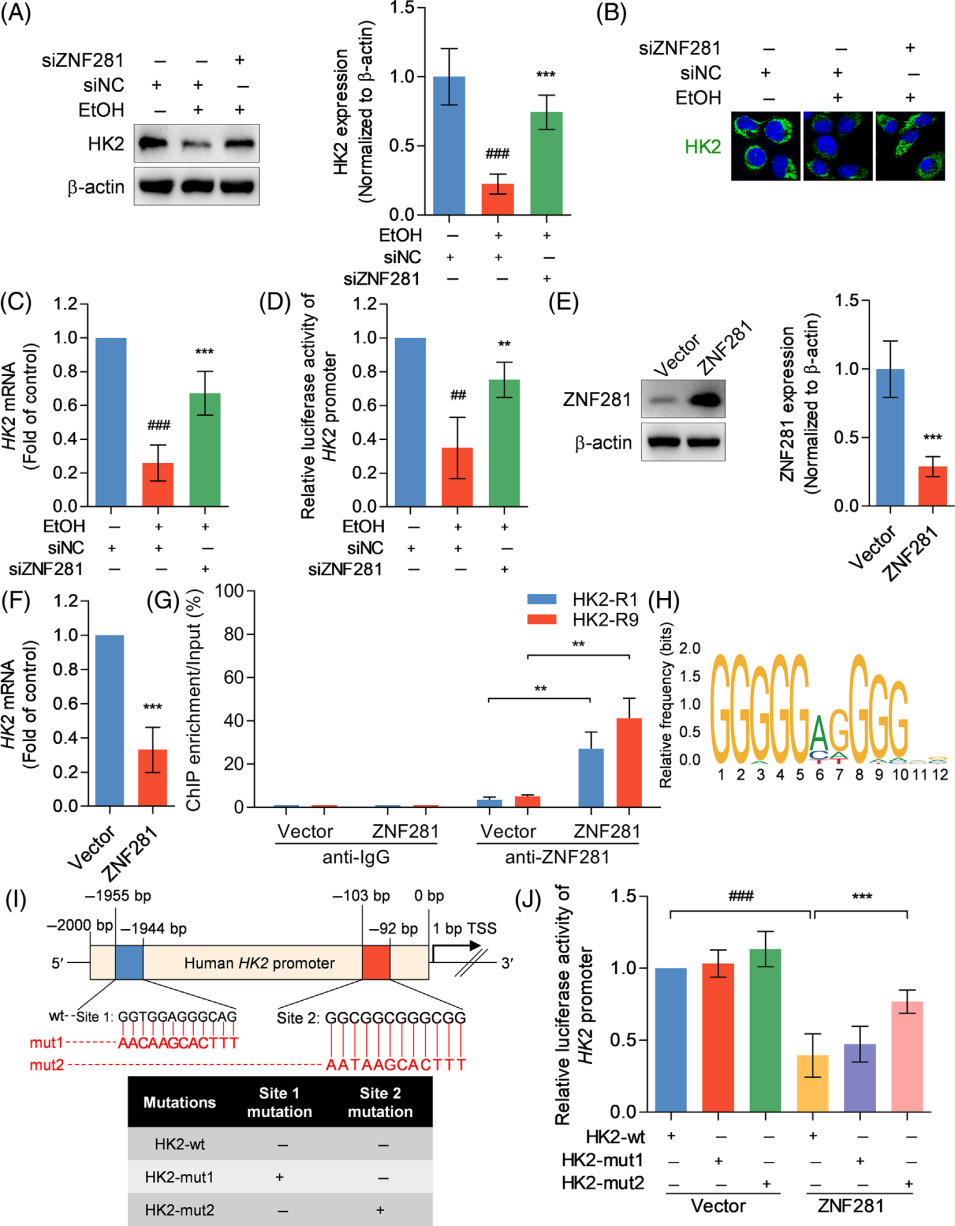
ZNF281 directly binds to HK2 promoter and initiates its transrepression. (A–D) HL‐7702 cells were transfected with siNC or siZNF281 for 24 h and treated with vehicle or 100‐mM ethanol for 24 h. (A, B) Intracellular HK2 protein abundance measured by immunoblots and immunofluorescence staining. (C) Intracellular HK2 mRNA abundance measured by qRT‐PCR. (D) HK2 promoter activity determined by dual‐luciferase reporter gene assay. ^##^ *p* < 0.01, ^###^ *p* < 0.001 versus siNC+vehicle. **p < 0.01, ***p < 0.001 versus siNC+100‐mM ethanol. (E–G) HL‐7702 cells were transfected with pcDNA3.1(+)‐ZNF281 recombinant plasmid or pcDNA3.1(+) empty vector for 24 h. (E) Intracellular ZNF281 protein abundance determined by immunoblots. (F) Intracellular HK2 mRNA abundance detected by qRT‐PCR. (G) HK2 enrichment after anti‐IgG or anti‐ZNF281 treatment determined by ChIP‐qRT‐PCR. **p < 0.01 versus vector. (H) JASPAR database showing the sequence motifs of the consensus binding sites of human ZNF281. (I) Schematic diagram showing putative ZNF281 binding sites in HK2 promoter and sequences of pGL3‐HK2‐wild‐type and mutant type plasmids. (J) HL‐7702 cells were transfected with ZNF281 recombinant plasmid or empty vector for 24 h and transfected with pGL3‐HK2‐wt, pGL3‐HK2‐mut1, or pGL3‐HK2‐mut2 plasmid for 48 h. HK2 promoter activity determined by dual‐luciferase reporter gene assay. ^###^ *p* < 0.001 versus vector+pGL3‐HK2‐wt. ***p < 0.001 versus ZNF281 plasmid+ pGL3‐HK2‐wt.

The molecular mechanisms underlying HK2 regulation by ZNF281 was investigated. Immunoblots showed that ethanol administration suppressed intracellular HK2 expression, while additional transfection with ZNF281 siRNA rescued HK2 expression (Figure 6A,B). qRT‐PCR showed ZNF281 interference also altered HK2 mRNA levels in hepatocytes under ethanol exposure (Figure 6C). Dual‐luciferase reporter gene assay showed that ZNF281 knockdown rescued ethanol‐damaged HK2 promoter activity in hepatocytes (Figure 6D). To specifically explore the regulatory mechanism of ZNF281, ZNF281 was overexpressed in hepatocytes and found to be effective in declining HK2 mRNA levels (Figure 6E,F). ChIP analysis revealed that anti‐ZNF281 caused ZNF281 protein enrichment to the 5′ regulatory region of HK2 gene after ZNF281 overexpression (Figure 6G). Then, JASPAR database was applied to predict potential binding sequences of ZNF281 to HK2 promoter region based on an available matrix and two putative binding sites were identified (Figure 6H). The two sites were dividually mutated to verify possible sequence motifs essential for ZNF281 binding. Dual‐luciferase reporter gene assay showed that the sequence of 5′‐GGCGGCGGGCGG‐3′ located within HK2 promoter region (−104 to −93 bp) was necessary for ZNF281 binding and regulate HK2 promoter activity (Figure 6I,J). In short, these data indicated that ZNF281 fell at a specific site within HK2 promoter region to transcriptionally repress HK2 expression.

### 3.7 ZNF281 silence ameliorates liver injury and hepatocyte senescence in alcohol‐fed mice

**FIGURE 7:**
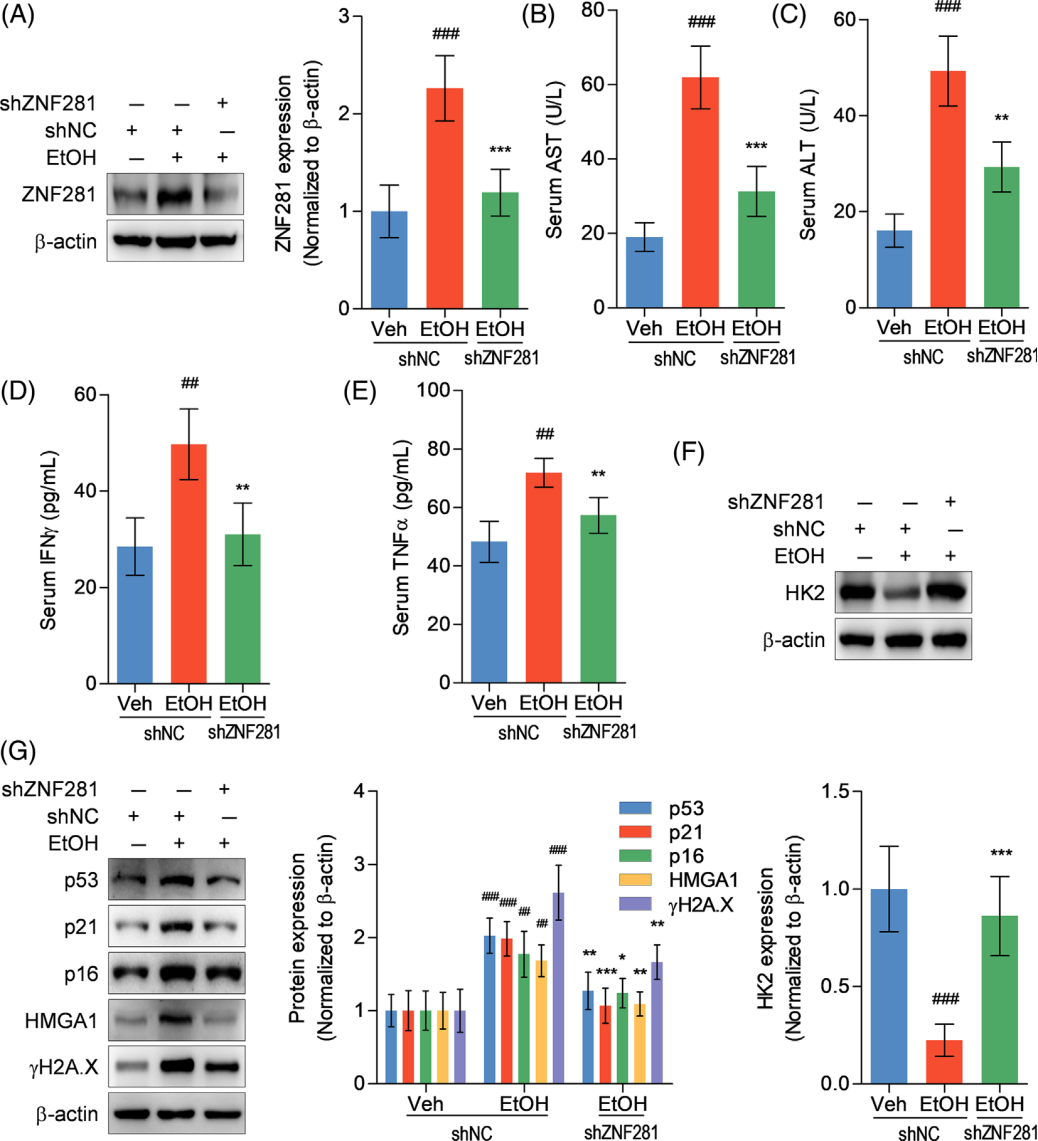
ZNF281 silence ameliorates liver injury and hepatocyte senescence in alcohol‐fed mice. Mice were transfected with adeno‐associated virus encoding shNC or shZNF281 through caudal vein and then treated with vehicle or alcohol by gavage for 4 weeks. (A) Hepatic ZNF281 protein abundance determined by immunoblots. (B, C) Serum AST and ALT activities. (D, E) Serum IFNγ and TNFα levels measured by ELISA. (F, G) Hepatic ZNF281, p53, p21, p16, HMGA1, and γH2A.X protein abundance determined by immunoblots. ^##^ *p* < 0.01, ^###^ *p* < 0.001 versus shNC+vehicle. *p < 0.05, **p < 0.01, ***p < 0.001 versus shNC+alcohol

According to the aggressive role of ZNF281 in causing ethanol‐induced hepatocyte senescence and cytotoxicity, we further determined whether ZNF281 deficiency could provide hepatoprotection against alcoholic liver disease in vivo. Immunoblots showed that ZNF281 shRNA remarkably reduced ZNF281 protein abundance in liver tissues of alcohol‐fed mice (Figure 7A). With alcohol gavage, serum AST and ALT activities were significantly elevated in wild‐type mice but fell back in ZNF281‐deficient mice (Figure 7B,C). ELISA results showed that serum proinflammatory cytokines IFNγ and TNFα levels were also decreased in ZNF281‐knockdown mice when compared with wild‐type mice (Figure 7D,E). HK2 protein abundance in liver tissues was reduced in mice with alcoholic liver disease, which was rescued by ZNF281 knockdown (Figure 7F). Also, the protein expression of p53, p21, p16, HMGA1, and γH2A.X was elevated in liver tissues from alcohol‐fed mice, which, however, were relatively diminished in alcohol‐fed ZNF281‐knockdown mice (Figure 7G). In summary, the results above suggested that ZNF281 deficiency relieves liver injury and hepatocyte senescence in mice with alcoholic liver disease.

## 4 DISCUSSION

ZNF281 is a zinc‐finger factor that is aberrantly expressed in various types of cancers and leads to cancer progression and metastasis.^12^, ^26^ In this study, the implication of ZNF281 was extended by discussing its role in alcoholic liver disease and our results suggested that high ZNF281 expression under ethanol exposure was a critical inducer of hepatocyte senescence and alcoholic liver injury.

According to our previous study, senescent cells were significantly multiplied in mice livers after four‐week alcohol consumption and in hepatocytes under 100‐mM ethanol exposure for 24 h.^5^, ^6^ In this study, we observed that ZNF281 expression was also increased in human hepatocytes and murine livers along with the progression of alcohol‐induced cell damage, which suggested an association between ZNF281 and alcohol‐induced hepatocyte senescence. Hepatocytes are prone to the hepatotoxicity of alcohol or its metabolic product acetaldehyde, which was featured by decreased cell viability and increased AST, ALT, and LDH release.^5^, ^6^ The current study further identified that ZNF281 deficiency could rescue hepatocytes from ethanol‐caused cell injury. Thus, ZNF281 could be a mediator of alcohol‐triggered cytotoxicity.

We next investigated whether ZNF281 was involved in ethanol‐induced hepatocyte senescence. We found that hepatocytes undergoing senescence, labelling as SA‐β‐gal‐positive cells, upon ethanol exposure were reduced when cellular ZNF281 was knockdown. Cellular senescence is characterized by cell cycle arrest and halted cell proliferation.^27^ Ethanol exposure‐caused cell cycle arrest at G0/G1 phase and cell viability decline were diminished when cellular ZNF281 expression was silent. Cellular senescence is predominantly mediated by cell cycle regulators p53, p21, and p16, wherein p21 and p16 are targets under p53 regulation.^28^, ^29^ P21 and p16 expression were enhanced by ethanol administration, which was consistent with the finding in our previous study.^5^ Upstream p53 expression was also enhanced upon ethanol stimulation, which was abolished when ZNF281 silence. P53 was also found to be upregulated in 48‐h palmitic acid‐incubated senescent AML12 murine hepatocytes.^30^ Intriguingly, ethanol exposure to HCC cells for 2 weeks caused significant cellular p53 reduction.^31^ In colorectal cancer cells, ectopic p53 expression could repress ZNF281 expression and negatively affect cancer progression.^32^ Accordingly, we postulated that the alternation of p53 and its association with ZNF281 could vary among different types and status of cells. Intriguingly, we found that ethanol insult provoked p53 protein translocation from nucleus into cytoplasm, which could be explained by the finding that p53 accumulated in perinuclei and colocalized in mitochondrial matrix and triggered mitochondrial permeability transition pore during oxidative stress.^33^ This speculation was also confirmed by our observation that mitochondrial ROS abundance was remarkably increased in hepatocytes after ethanol stimulation. Of note, mitochondrial ROS‐induced oxidative stress has been considered accelerating cellular senescence.^34^ Our further study showed that ZNF281 silence not only neutralized ethanol‐induced activation of p53‐p21/p16 senescent signalling but also prevented mitochondrial ROS overproduction. γH2A.X is a vital marker indicating DNA breaks.^35^ In this study, we observed that ethanol caused an increase in the protein abundance of γH2A.X, whereas ZNF281‐knockdown hepatocytes were resistant to this alternation, suggesting that ZNF281 could be required for ethanol‐triggered DNA breaks in hepatocytes. Notably, a previous contradictory study showed that ZNF281 was recruited to DNA breaks to facilitate DNA repair in tumour cells.^36^ Herein, the function of ZNF281 in modulating DNA breaks and repair under distinct conditions could be heterogeneous. Damaged or aged mitochondria in senescent cells are deficient in their capacity to metabolize fatty acids, which generally results in intracellular accumulation of lipid droplets.^22^ Consistently, this study verified that ethanol not only initiated mitochondrial fragmentation but also induced hepatosteatosis. Of note was that ZNF281 deficiency exerted robust protection against senescence‐associated hepatocyte damage.

A mitochondrial quality control system involves a series of processes including mitochondrial biogenesis, mitophagy, mitochondrial proteostasis, and mitochondria‐mediated cell death.^37^ Mitophagy can scavenge damaged or aged mitochondria through lysosome‐dependent degradation pathway.^15^, ^38^ Previously, we and Zhou et al. found that mitophagy was impaired in ethanol‐treated hepatocytes.^20^, ^39^ PINK1 facilitates depolarization‐induced Parkin translocation onto mitochondria, thus promoting mitophagy and reducing functionally abnormal mitochondria.^40^ Consistent results were obtained in this work regarding reduced PINK1 and Parkin protein abundance, decreased recruitment of Parkin to mitochondrial outer membrane and specifically PINK1, and decreased colocalization between mitochondria and lysosomes in ethanol‐treated hepatocytes. Furthermore, knockdown of ZNF281 rescued PINK1/Parkin‐dependent mitophagy in ethanol‐exposed hepatocytes. Intriguingly, ZNF281 interference caused p62 deposition and LC3 declination, which triggered autophagy disorder in colorectal cancer cells.^16^ This discrepancy could possibly be associated with the double‐edged property of autophagic machinery and the different states of cells.

HK2 is a predominant HK isoform localized in mitochondrial outer membrane. HK2 is a substrate of Parkin and can recruit Parkin to mitochondria and launches mitophagy.^41^ Herein, we found that HK2 protein abundance was reduced in the mitochondrial fraction from ethanol‐treated hepatocytes and the whole cells, suggesting that less HK2 localization in mitochondria and low HK2 protein expression under ethanol exposure. However, ZNF281 knockdown rescued cellular HK2 expression and its recruitment to mitochondria. HK2 silence did not alter the expression of PINK1 and Parkin but reduced PINK1/Parkin complex, which laterally confirmed the results from a recent study that mitophagy was inhibited when HK2 dissociation from mitochondria.^25^ Our findings suggested that HK2 protein was required for PINK1/Parkin‐dependent mitophagy in hepatocytes and was negatively regulated by ZNF281. Intriguingly, a previous study showed that HK2 separation from mitochondria triggered Parkin‐mediated mitophagy that was independent of PINK1 pathway in response to ischemia.^42^ This divergence could be due to the functionality of HK2 itself, since HK2 promoted assembly of high‐molecular weight complex of PINK1 and phosphorylation of ubiquitin in response to mitochondrial damage.^43^

ZNF281 is a classical transcription factor and has activation and repression roles in gene transcription.^44^ In this study, we found that HK2 expression was negatively regulated by ZNF281 at both transcriptional and translational levels. Further investigations identified that the motif of 5′‐GGCGGCGGGCGG‐3′ within HK2 promoter region was required for ZNF281 to bind to and transrepress HK2 expression.

Finally, in vivo effects of ZNF281 on alcoholic liver damage was evaluated. Systematic ZNF281 knockdown was realized in mice using adenovirus‐associated virus vectors encoding shZNF281 injection. On this basis, we observed that ZNF281‐deficient mice were resistant to alcohol‐caused liver injury and hepatocyte senescence, which further verified the results from in vitro study.

**FIGURE 8:**
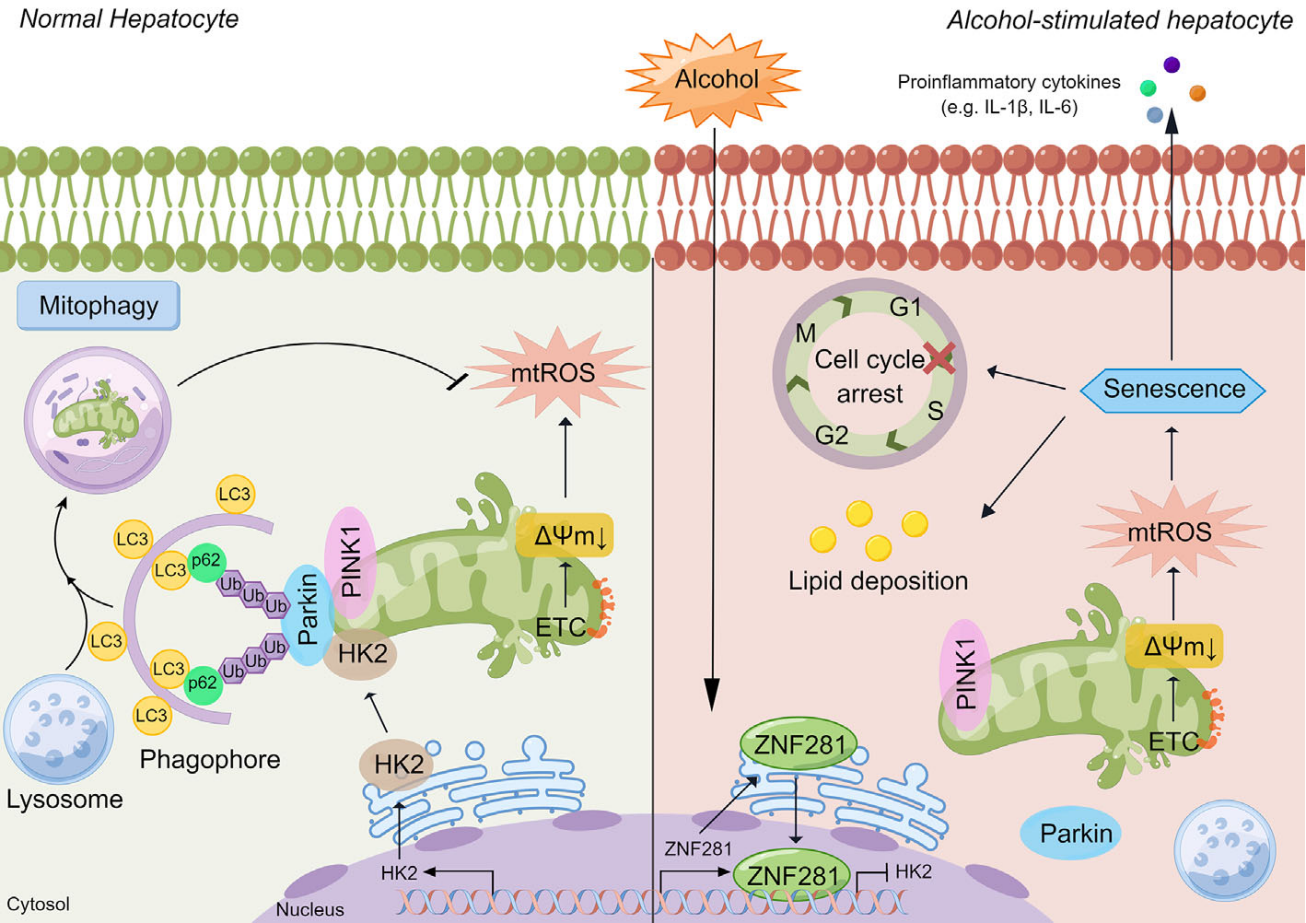
Scheme of the molecular mechanisms underlying ethanol‐caused hepatocyte senescence (By Figdraw). Under physiological conditions, damaged mitochondria can be removed and recycled via PINK1/Parkin‐mediated mitophagy, maintaining cellular homeostasis. Under pathological conditions like ethanol exposure, electron transport chain (ETC) breaks are enhanced and mitochondrial membrane potential is declined, inducing oxidative stress and lipid peroxidation. Ethanol also induces ZNF281 expression in hepatocytes, which translocates to nucleus to serve as a transcriptional repressor of HK2. Absence of HK2 decreased PINK1‐Parkin interaction and mitophagy process, causing accumulation of damaged mitochondria. Afterwards, cellular senescence was provoked with the characteristics of cell cycle arrest and SASP, promoting the progression of alcoholic liver disease.

In conclusion, ZNF281 is lowly expressed in normal hepatocytes. When stimulated with alcohol, ZNF281 expression in hepatocytes was highly upregulated, which serves as a repressor and transcriptionally suppresses HK2 expression, destabilizes HK2‐mediated PINK/Parkin‐dependent mitophagy, and turns cells towards senescence (Figure 8). Targeting at modulating ZNF281 could be anticipated to be a potential therapeutic strategy for alcoholic liver disease.

## CONFLICT OF INTEREST

The authors declare that there are no conflicts of interest.

## Supporting information


**Table S1.** Primers used in qRT‐PCR for determining the expression of interested genes in HL‐7702 human hepatocytes.

**Table S2.** Primers used in ChIP‐qRT‐PCR for determining putative binding region of ZNF281 protein on HK2 promoter.

**Table S3.** Primers used in site‐directed mutagenesis for mutating DNA motifs on HK2 promoter.

## Data Availability

The data that support the findings of this study are available from the corresponding author upon reasonable request.
