# Supplementary material for: ZNF281 drives hepatocyte senescence in alcoholic liver disease by reducing HK2‐stabilized PINK1/Parkin‐mediated mitophagy
Source: Cell Prolif. 2022 Dec 14;56(3):e13378. doi: 10.1111/cpr.13378 (PMC9977663; doi:10.1111/cpr.13378)
Supplement: Supplementary file 1 — Table S1. Primers used in qRT‐PCR for determining the expression of interested genes in HL‐7702 human hepatocytes. [file CPR-56-e13378-s001.docx]

**Table S1 Primers used in qRT-PCR for determining the expression of interested genes in HL-7702 human hepatocytes**

| Genes | Primer sequences (5’–3’) | |
| --- | --- | --- |
| *ZNF281* | Forward | AGTGTGGTTTCGGCCAATCT |
|  | Reverse | CTGGGAAGGTGTCAACTGCT |
| *HK2* | Forward | ACGCCAAAATCACGTCTCCG |
|  | Reverse | AGAGATACTGGTCAACCTTCTGC |
| *GAPDH* | Forward | AATGGGCAGCCGTTAGGAAA |
|  | Reverse | GCCCAATACGACCAAATCAGAG |
