# Supplementary material for: ZNF281 drives hepatocyte senescence in alcoholic liver disease by reducing HK2‐stabilized PINK1/Parkin‐mediated mitophagy
Source: Cell Prolif. 2022 Dec 14;56(3):e13378. doi: 10.1111/cpr.13378 (PMC9977663; doi:10.1111/cpr.13378)
Supplement: Supplementary file 2 — Table S2. Primers used in ChIP‐qRT‐PCR for determining putative binding region of ZNF281 protein on HK2 promoter. [file CPR-56-e13378-s003.docx]

**Table S2** **Primers used in ChIP-qRT-PCR for determining putative binding region of ZNF281 protein on HK2 promoter**

| Genes | Primer sequences (5’–3’) | | Product size (bp) |
| --- | --- | --- | --- |
| *HK2-R1* | Forward | GTTTCTCCTCAGGGAGTTTGAAACTG | 200 |
|  | Reverse | AGTTAATCACATCCTCCATCTGGAC |  |
| *HK2-R2* | Forward | GTCCTCAACATCCCTGAGAGAAG | 212 |
|  | Reverse | AGCTGGGATTACAGATGTGCTC |  |
| *HK2-R3* | Forward | ATTTGGGACGCTGAAGTGGG | 225 |
|  | Reverse | GCTGTCTGCAATGTGTACAGC |  |
| *HK2-R4* | Forward | ACCCCACTGTTTGAAAAACTCCTCC | 201 |
|  | Reverse | AGCTAATGCTAGACACCAGACTCCA |  |
| *HK2-R5* | Forward | GGGGTTGGAGCTTCCACTCCTCTCA | 206 |
|  | Reverse | GGCCCTATTCGCTTGCCCCATTATCC |  |
| *HK2-R6* | Forward | TTAAAGTAGAGGGACAAGGGTTCTC | 236 |
|  | Reverse | AAATAAATCAGCCGGACATGGTG |  |
| *HK2-R7* | Forward | ATTTTTATTTGGGAAGATGGGGGTC | 282 |
|  | Reverse | GCATCCTAGTTCTTACCTATTGTAGCA |  |
| *HK2-R8* | Forward | TTTAGACGTGTGACTGGGC | 199 |
|  | Reverse | TAAAGGCTGGGACGGAAAG |  |
| *HK2-R9* | Forward | GCCACGGACCACACGTCCCATC | 240 |
|  | Reverse | GGCTTATTGAGGGGCTGCCGGCC |  |
