# Supplementary material for: ZNF281 drives hepatocyte senescence in alcoholic liver disease by reducing HK2‐stabilized PINK1/Parkin‐mediated mitophagy
Source: Cell Prolif. 2022 Dec 14;56(3):e13378. doi: 10.1111/cpr.13378 (PMC9977663; doi:10.1111/cpr.13378)
Supplement: Supplementary file 3 — Table S3. Primers used in site‐directed mutagenesis for mutating DNA motifs on HK2 promoter. [file CPR-56-e13378-s002.docx]

**Table S3 Primers used in site-directed mutagenesis for mutating DNA motifs on HK2 promoter**

| Genes | Primer sequences (5’–3’) | |
| --- | --- | --- |
| *HK2-mut1* | Forward | AACAAGCACTTTCCCCGAATACCCCAGC |
|  | Reverse | GGAAAGTGCTTGTTCTCTTCTCTGAACTAGTTACAGTTTC |
| *HK2-mut2* | Forward | CACAATAAGCACTTTTCCGTGGGCGCACA |
|  | Reverse | AAAAGTGCTTATTGTGGCGCAGCCAATCATC |
